# Evaluation of Pyridoacridine Alkaloids in a Zebrafish Phenotypic Assay

**DOI:** 10.3390/md8061769

**Published:** 2010-06-02

**Authors:** Xiaomei Wei, Tim S. Bugni, Mary Kay Harper, Imelda T. Sandoval, Elizabeth J. Manos, Jennifer Swift, Ryan M. Van Wagoner, David A. Jones, Chris M. Ireland

**Affiliations:** 1 Department of Medicinal Chemistry, University of Utah, Salt Lake City, UT84112, USA; E-Mails: xiaomei.wei@pharm.utah.edu (X.M.W.); m.k.harper@pharm.utah.edu (M.K.H.); r.m.vanwagoner@utah.edu (R.M.V.W.); 2 Department of Oncological Sciences, Huntsman Cancer Institute, 2000 Circle of Hope, Salt Lake City, UT84112, USA ; E-Mails: Imelda.Sandoval@hci.utah.edu (I.T.S.); Betsy.Manos@hci.utah.edu (E.J.M.);Jennifer.Swift@hci.utah.edu (J.S.) ; David.Jones@hci.utah.edu(D.A.J.)

**Keywords:** pyridoacridine alkaloids, *Xestospongia* cf. *carbonaria*, zebrafish

## Abstract

Three new minor components, the pyridoacridine alkaloids 1-hydroxy-deoxyamphimedine (**1**), 3-hydroxy-deoxyamphimedine (**2**), debromopetrosamine (**3**), and three known compounds, amphimedine (**4**), neoamphimedine (**5**) and deoxyamphimedine (**6**), have been isolated from the sponge *Xestospongia* cf*. carbonaria*, collected in Palau. Structures were assigned on the basis of extensive 1D and 2D NMR studies as well as analysis by HRESIMS. Compounds **1**–**6** were evaluated in a zebrafish phenotype-based assay. Amphimedine (**4**) was the only compound that caused a phenotype in zebrafish embryos at 30 μM. No phenotype other than death was observed for compounds **1**–**3**, **5**, **6.**

## 1. Introduction

Zebrafish have been identified as a valuable whole animal platform for phenotypic screening at various stages of the drug discovery process [1–4]. Some of the advantages that zebrafish offer in laboratory assays include the transparency of their embryos, allowing direct observation of organ morphology during treatment or visualization of specific molecular species using hybridization techniques; rapid development and short reproductive cycles; and easy maintenance in 96-well plates. Although the potential value of zebrafish in screening natural product extract libraries has been noted [5,6 ], to date relatively few zebrafish-based natural product screening programs, from either plants or marine sources, have been reported [7–9]. As part of a pilot study, we screened a prefractionated extract library [10] from marine invertebrates for zebrafish phenotypic activity. This small library included four fractions from each of forty organisms that were selected based on a known high incidence of cytotoxic compounds in their extracts. The rationale behind organism selection was to determine if our fractionation method is effective at enabling observation of phenotypic activity in the presence of strong background cytotoxicity. Within the 160 fractions, 25% exhibited embryo toxicity whereas an additional 8% caused various other phenotypic responses 24 hours after treatment. We report here the results from one of the organisms displaying activity, the sponge *Xestospongia* cf. *carbonaria* collected in Palau, which caused a phenotype of abnormal notochord development and death in the primary screen. Three new pyridoacridine alkaloids **1**–**3** as well as the known compounds **4**–**6**, were isolated from a large scale extraction of this sponge. The pure compounds **1**–**6** were evaluated in the zebrafish assay, identifying amphimedine (**4**) as the agent responsible for the phenotypic response in the primary screen.

## 2. Results and Discussion

The specimen of *Xestospongia* cf. *carbonaria* was extracted with MeOH. The crude extract was separated on HP20SS resin according to a prefractionation protocol previously reported by this lab [11]. Subsequent bioassay-guided fractionation resulted in the isolation of the pyridoacridine alkaloids **1**–**3** as their respective trifluoroacetate salts, along with the known compounds amphimedine (**4**), neoamphimedine (**5**), and deoxyamphimedine (**6**). 1-Hydroxy-deoxyamphimedine (**1**) was obtained as a red-orange amorphous solid. A molecular ion in the positive HRESIMS spectrum at *m/z* 314.0945 corresponded to a molecular formula of C_19_H_12_N_3_O_2_ (Δ 4.8 ppm), which was isomeric with amphimedine (**4**) and neoamphimedine (**5**). The strong absorption bands at 3382 and 1687 cm^−1^ in the IR spectrum indicated that **1** contained a hydroxyl group and a conjugated carbonyl group. The structure of **1** was elucidated by interpretation of NMR data (Table 1) and comparison with spectral data for deoxyamphimedine (**6**). The only difference observed in the NMR data between **1** and **6** was in the A ring system. For **1**, two doublets and one doublet of doublets at δ_H_ 7.48 (*J* = 8.0 Hz), 8.45 (*J* = 8.0 Hz) and 7.91 (*J* = 8.0, 8.0 Hz) ppm, respectively, indicated an additional hydroxyl present at either C-1 or C-4. The position of the hydroxyl group was identified as C-1 based on a NOESY correlation between signals at δ_H_ 8.45 (H-4) and 9.13 (H-5) ppm. The gHMBC spectrum also supported the assignment of **1** as 1-hydroxy-deoxyamphimedine.

A molecular formula of C_19_H_12_N_3_O_2_ for 3-hydroxy-deoxyamphimedine (**2**) was consistent with both the HRESIMS (*m/z* 314.0938, [M]^+^, Δ 2.5 ppm) and with proton and carbon counts in the respective NMR spectra. Compound **2** is isomeric with **1** and showed a very similar IR spectrum suggesting the presence of a hydroxyl group (ν_max_ 3421 cm^−1^) and an iminoquinone (ν_max_ 1685 cm^−1^). ^1^H chemical shifts and coupling patterns for the A ring were different from those of **1**. The ^1^H NMR spectrum of **2** contained a broad doublet at δ_H_ 8.20 ppm (H-4, *J* = 2.5 Hz) and a doublet of doublets at δ_H_ 7.63 ppm (H-2, *J* = 9.0, 2.5 Hz) indicating that the hydroxyl was at C-3. Thus, the A ring in **2** was identified as being a 1, 3, 4-trisubstituted benzene, which marks the difference between **1** and **2.** A 1D NOESY experiment also showed a correlation between signals at δ_H_ 8.20 (H-4) and 9.00 ppm (H-5) in **2**, thus confirming the identity of **2** as 3-hydroxy-deoxyamphimedine.

The structure of debromopetrosamine (**3**) appeared in a review on marine pyridoacridine alkaloids [12], but the compound has never been formally described. The alkaloid **3** was isolated as a purple-blue amorphous solid and determined to have a molecular formula of C_21_H_18_N_3_O_2_ by HRESIMS (*m/z* 344.1415, [M]^+^, Δ 4.6 ppm). A strong and broad absorption band at 1682 cm^−1^ in the IR spectrum suggested that **3** contained multiple conjugated ketones. The gHSQC spectrum and ^1^H NMR data indicated seven aromatic protons, two methylene protons (δ_H_ 4.41, H-6) and nine *N*-methyl protons (δ_H_ 4.53, Me-14 and δ_H_ 3.84, Me-15, 16). The ^1^H chemical shifts and coupling patterns of ring A in **3** were nearly identical with those of amphimedine (**4**) and neoamphimedine (**5**). Ortho-coupled proton resonances at δ_H_ 8.75 (H-11) and δ_H_ 9.39 (H-12), along with a low-field proton at δ_H_ 9.72 (H-9) indicated **3** contained the same E ring pattern as deoxyamphimedine (**6**) although the ^1^H and ^13^C chemical shifts of Me-14 showed it was not attached to a quaternary nitrogen. The degenerate *N,N-*dimethyl proton singlets at δ_H_ 3.84 showed gHMBC correlations to an *N*-methyl carbon at δ_C_ 54.1, an aromatic quaternary carbon at δ_C_ 115.1 (C-7a), the methylene carbon at δ_C_ 71.4 (C-6) and a weak long-range correlation to δ_C_ 187.2 (C-5). Additionally, the gHMBC spectrum showed correlations from methylene protons δ_H_ 4.41 (H-6) to the degenerate *N*-methyl carbons C-15 and C-16 (δ_C_ 54.1), a quaternary aromatic carbon at δ_C_ 115.8 (C-4b), as well as the carbonyl at δ_C_ 187.2 (C-5). The gHMBC correlations between H-4 and C-4b; H-9 and C-8; H-11 and C12a; and H12 to C12b and C-8a elucidated the connectivities of the A, B, C, and D rings. COSY correlations from δ_H_ 9.72 (H-9) to δ_H_ 8.75 (H-11); from δ_H_ 9.27 (H-4) to δ_H_ 7.84 (H-3); from δ_H_ 8.19 (H-1) to δ_H_ 7.84 (H-3) and δ_H_ 7.73 (H-2); from δ_H_ 4.41 (H -6) to δ_H_ 3.84 (H -15, H-16); and from δ_H_ 8.75 (H-11) to δ_H_ 9.39 (H -12) and δ_H_ 4.53 (H -14) further confirmed the carbon connections. Based on these assignments and comparison of spectral data with those from the known compounds petrosamine [13] and petrosamine B [14], we conclude that **3** is debromopetrosamine.

**Figure 2:**
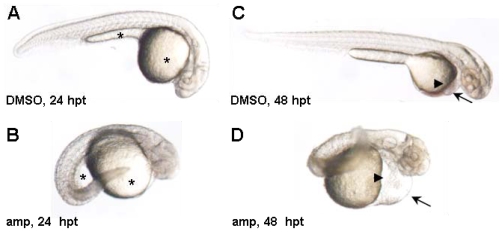
Amphimedine-treated embryos exhibit heart, yolk and body shape defects. Treatment of embryos seven hours post-fertilization (hpf) with 30 μM amphimedine (amp) resulted in pericardial edema (arrow), extended heart (arrow head), enlarged yolk with thin extension (*), curving of the body and general necrosis at 24 hours post-treatment (hpt; B) and 48 hpt (D) compared to the DMSO-treated controls (A and C, respectively).

**Figure 3:**
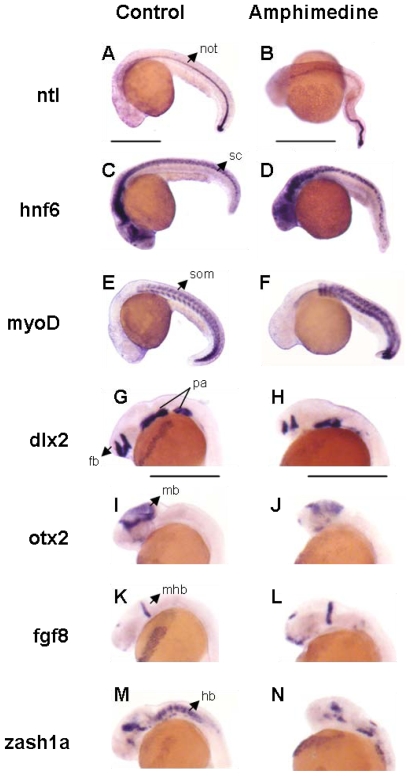

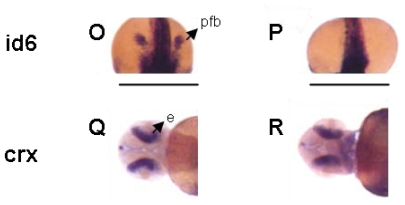
Additional developmental defects resulting from amphimedine treatment. *In situ* hybridization was performed on control (A, C, E, G, I, K, M, O, Q) and treated (B, D, F, H, J, L, N, P, R) embryos harvested at 24 h (*ntl*, *hnf6*, *myoD*, *dlx2*, *otx2*, *fgf8*, *zash1a*, *id6*) or 48 h (*crx*) post treatment. Scale bars, 50 μM. not, notochord; sc, spinal cord; som, somites; pa, pharyngeal arches; fb, forebrain; mb, midbrain; mhb, mid/hindbrain boundary; hb, hindbrain; pfb, pectoral fin bud; e, eye.

Of the pure compounds (**1**–**6**) isolated from this sponge, only amphimedine (**4**) showed a phenotype in the zebrafish assay at 30 μM. Embryos exposed to amphimedine (**4**) exhibited necrosis, pericardial edema and an enlarged yolk with thin extension (Figure 2B, D). The embryos also appeared short and grainy, with an extended heart, weak heartbeat, no circulation, and irregular curvature of the tail (Figure 2B, D). In order to further characterize the phenotypes induced by amphimedine (**4**), a series of *in situ* hybridization experiments were carried out that utilized digoxigenin-labeled antisense RNA probes against various regulatory genes involved in development and differentiation. These studies enabled selective high-contrast imaging of various organs. Treated embryos showed wavy notochord, spinal cord and abnormally shaped somites after imaging of *ntl*, *hnf6* and *myoD*, respectively (Figure 3B, D, F). They also lack pectoral fin buds, have slightly smaller eyes and shortened brain regions as shown by the imaging of known brain markers such as *dlx2*, *otx2*, *fgf8* and *zash1a* (Figure 3H, J, L, N, P, R). The variety and severity of phenotypic responses induced by amphimedine (**4**) suggest interference with a fundamental process in embryonic development or action against multiple systems. Unfortunately, at this time it is not possible to infer from the pattern of activities observed which specific targets amphimedine (**4**) might be modulating.

Since the first pyridoacridine, amphimedine (**4**) was identified in 1983 [15], over a hundred pyridoacridine alkaloids have been isolated from marine sources [16]. The bioactivity most often reported for pyridoacridine molecules has been cytotoxicity [17–22], although a wide array of other biological activities have also been documented, such as antimicrobial [23,24], anti-viral [25], and DNA-directed activities [26]. Neoamphimedine (**5**), a known topoisomerase II inhibitor [17], was not active in the zebrafish screen, which is consistent with our observations that other known top2 inhibitors, including etoposide, daunorubicin and doxorubicin show no phenotype, illustrating both the selectivity of the assay and the remarkable versatility of the pyridoacridine family. From a structure activity viewpoint, it is clear that minor structural differences can have a marked effect on the biological activity of the pyridoacridine class of compounds. It is worth noting that amphimedine (**4**), despite its low abundance relative to the toxic pyridoacridines also present in the extract, displayed observable activity in fairly crude fractions. This suggests that modest fractionation schemes that can be carried out in parallel with many extracts at low cost can be sufficient to allow detection of compounds causing interesting phenotypes, even in the presence of cytotoxic compounds.

## 3. Experimental Section

### 3.1. General Experimental Procedures

UV spectra were acquired in spectroscopy grade MeOH using a Hewlett-Packard 8452A diode array spectrophotometer. IR spectra were recorded on a JASCO FT/IR-420 spectrometer. NMR data for compounds **1**–**3** were acquired using a Varian INOVA 500 (^1^H 500 MHz, ^13^C 125 MHz) NMR spectrometer with a 3mm Nalorac MDBG probe or Varian INOVA 600 (^1^H 600 MHz, ^13^C 150 MHz) NMR spectrometer with a 5 mm Cold Probe for compound **2** referenced to residual solvent (δ_H_ 2.49, δ_C_ 39.51 for DMSO-*d_6_*; δ_H_ 3.30, δ_C_ 49.15 for CD_3_OD; and δ_H_ 1.94, δ_C_ 118.69 for CD_3_CN). High-resolution ESIMS analyses were performed on a Micromass Q-tof micro. Initial purification was performed on HP20SS resin. HPLC was performed on an Agilent 1100 system using Luna Phenyl-Hexyl (Phenomenex, Inc.) (250 × 10 mm, 5 μm; 250 × 4.6 mm, 5 μm) and Luna C_18_ (Phenomenex, Inc.)(250 × 4.6 mm, 5 μm) columns.

### 3.2. Biological Material

In order to obtain sufficient quantity of metabolites for biological testing, a large-scale collection of the source sponge was conducted. This sponge is identical to the material, *Xestospongia* cf. *carbonaria,* from which we previously reported the isolation of neoamphimedine [19]. This sponge is readily distinguished by its unique growth form, dark green staining pigmentation, and habitat. There has been confusion over its taxonomic placement, and it appears in the literature under several genus names including *Amphimedon, Axinyssa, Neopetrosia, Pellina,* and *Xestospongia*. Further investigation is needed to determine the correct taxonomic assignment of this sponge.

### 3.3. Extraction and Isolation

The *Xestospongia* cf*. carbonaria* specimen (1.206 kg) was extracted with 100% MeOH (3 × 1.8 L) to yield 54.26 g of crude extract. The crude extract was adsorbed on a 450 × 75 mm column packed with 80 g HP20SS resin that was sequentially eluted with 2 L each of water (FW), 25% (F1), 50% (F2), 75% (F3), and 100% (F4) IPA in water followed by 100% MeOH (F5). Fraction F1 (1.06 g) was partitioned between CHCl_3_ (3 **×** 200 mL) and 70% MeOH (aq; 200 mL). The dried aqueous MeOH phase was further fractionated by HPLC phenyl-hexyl employing MeOH/aqueous TFA (0.1%) gradients resulting in the isolation of compound **1** (1.0 mg), **2** (0.8 mg), **3** (3.0 mg), and deoxyamphimedine (**6**, 10.1 mg). Purification of the HP20SS F2 fraction yielded amphimedine (**4**, 1.2 mg) and neoamphimedine (**5**, 5.1 mg). Compounds **4**–**6** were identified by comparison to known standards.

*1-Hydroxy-deoxyamphimedine* (**1**): Trifluoroacetate salt; red-orange amorphous solid; UV (MeOH) λ_max_ (log ɛ) 204 (4.51), 246 (4.35), 286 (sh, 4.16), 392 (3.92) nm; IR (film) ν_max_ 3382, 2925, 2854, 1687, 1598, 1507, 1206, 1136, 801, 723 cm^−1^; ^1^H NMR and ^13^C NMR data, see Table 1; HRESIMS *m/z*314.0945 [M] ^+^(calcd for C _19_H_12_N_3_O_2_, 314.0930).

*3-Hydroxy-deoxyamphimedine* (**2**): Trifluoroacetate salt; orange-yellow amorphous solid; UV (MeOH) λ_max_ (log ɛ) 206 (4.95), 244 (4.94), 394 (3.34), 488 (3.24) nm; IR (film) ν_max_ 3421, 2925, 1685, 1505, 1443, 1210, 1139, 844, 803, 724 cm^−1^; ^1^H NMR and ^13^C NMR data, see Table 1; HRESIMS *m/z*314.0938 [M] ^+^(calcd for C _19_H_12_N_3_O_2_, 314.0930).

*Debromopetrosamine* (**3**): Trifluoroacetate salt; purple-blue amorphous solid; UV (MeOH) λ_max_ (log ɛ) 216 (4.49), 282 (4.74), 374 (3.38), 592 (3.15) nm; IR (film) ν_max_ 3063, 1682, 1648, 1588, 1498, 1206, 1129, 801, 722 cm^−1^; ^1^H NMR and ^13^C NMR data, see Table 1; HRESIMS *m/z* 344.1415 [M]^+^ (calcd for C_21_H_18_N_3_O_2_, 344.1399).

### 3.4. Zebrafish Screen

*Danio rerio* (zebrafish) were maintained as previously described [27]. Fertilized embryos were collected following natural spawnings in 2X PTU (1X E3 medium, 30.4 mg/L phenylthiouera). Embryos were periodically checked for death and developmental delay. At seven hours post-fertilization (hpf), embryos were arrayed in 96-well plates at 1 embryo/well. Pure compounds were then added to the desired concentration, with dimethyl sulfoxide (DMSO) as vehicle control. DMSO was kept at 0.5% of the total assay volume. Embryos were grown at 28.5 °C and examined visually with a dissecting microscope at 1, 2, 3 and 6 days post treatment. Early toxicity was noted by examining the embryos at 1, 5, and 17 hours post treatment. Embryos were photographed live. All experiments were repeated at least twice, in duplicate.

*In situ* hybridizations were performed as previously described using digoxigenin-labeled riboprobes for *crx* (cone rod homeobox), *dlx2* (distal-less homeobox protein 2), *fgf8* (fibroblast growth factor 8), *hnf6* (hepatocyte nuclear factor-6), *id6* (inhibitor of DNA-binding/differentiation 6), *myoD* (myogenic differentiation1), *ntl* (no tail), *otx2* (orthodentical homeobox protein 2) and *zash1a* (zebrafish achaete/scute homologue 1a) [28]. Embryos were photographed with an Olympus SZX12 digital camera.

## Figures and Tables

**Figure 1:**
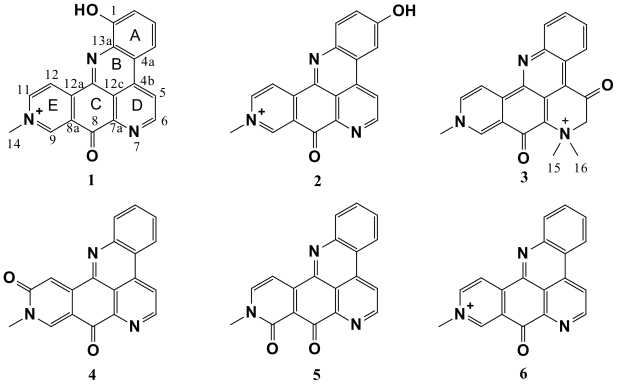
Structures of compounds **1**–**6**.

**Table 1:** ^1^H and ^13^C-NMR data of pyridoacridine alkaloids (**1**–**3**).

Posn.	1			2			3		
	
	δ_H_, mult (*J*_H-H_)	δ_C_, mult a		δ_H_, mult (*J*_H-H_)	δ_C_, mult b		δ_H_, mult (*J*_H-H_)	δ_C_, mult c	
1		156.1	C	8.39, d (9.0)	135.8	CH	8.19, d (9.0)	143.0	CH
2	7.48, d (8.0)	116.5	CH	7.63, dd (9.0, 2.5)	124.3	CH	7.73, dd (9.0, 7.5)	128.4	CH
3	7.91, dd (8.0, 8.0)	133.3	CH		162.7	C	7.84, dd (8.0, 7.5)	133.5	CH
4	8.45, d (8.0)	114.5	CH	8.20, d (2.5)	108.0	CH	9.27, d (8.0)	125.1	CH
4a		123.8	C		126.6	C		125.2	C
4b		137.9	C		140.9	C		115.8	C
5	9.13, d (6.0)	121.8	CH	9.00, d (6.0)	122.9	CH		187.2	C
6	9.37, d (6.0)	150.2	CH	9.30, d (6.0)	151.4	CH	4.41, s	71.4	CH_2_
7a		147.0	C		149.5	C		115.1	C
8		180.0	C		180.6	C		161.0	C
8a		130.3	C		130.8	C		132.7	C
9	9.87, s	146.0	CH	9.82, s	147.9	CH	9.72, s	145.9	CH
11	9.40, d (6.5)	147.8	CH	9.17, d (6.5)	148.7	CH	8.75, d (6.5)	142.0	CH
12	9.90, d (6.5)	123.5	CH	9.38, d (6.5)	124.1	CH	9.39, d (6.5)	122.9	CH
12a		147.9	C		149.2	C		143.5	C
12b		143.1	C		145.5	C		139.5	C
12c		120.1	C		120.8	C		129.9	C
13a		134.4	C		141.5	C		143.1	C
14	4.57, s	48.3	CH_3_	4.58, s	49.3	CH_3_	4.53	49.0	CH_3_
15							3.84, s (3H)	54.1	CH_3_
16							3.84, s (3H)	54.1	CH_3_

a ^13^C NMR data assigned based on the HSQC and HMBC in DMSO-*d_6_* (500 MHz).

b ^13^C NMR data assigned based on the HSQC and HMBC in CD_3_OD (600 MHz).

c ^13^C NMR data assigned based on the HSQC and HMBC in CD_3_CN (500 MHz).

## References

[1] Bowman TV Zon LI Swimming into the future of drug discovery: *in vivo* chemical screen in zebrafish ACS Chem. Biol 2010 5 159 161 20166761 10.1021/cb100029t PMC4712380

[2] Chakraborty C Hsu CH Wen ZH Lin CS Aqoramoorthy G Zebrafish: a complete animal model for *in vivo* drug discovery and development Curr. Drug Metab 2009 10 116 124 19275547 10.2174/138920009787522197

[3] Kaufman CK White RM Zon LI Chemical genetic screening in the zebrafish embryo Nat. Protoc 2009 4 1422 1432 19745824 10.1038/nprot.2009.144 PMC2943144

[4] Pichler FB Laurenson S Williams LC Dodd A Copp BR Love DR Chemical discovery and global gene expression analysis in zebrafish Nat. Biotechnol 2003 21 879 883 12894204 10.1038/nbt852

[5] Mandrekar N Thakur NL Significance of the zebrafish model in the discovery of bioactive molecules from nature Biotechnol. Lett 2009 31 171 179 18931972 10.1007/s10529-008-9868-1

[6] Crawford AD Esquerra CV de Witte PA Fishing for drugs from nature: zebrafish as a technology platform for natural product discovery Planta Med 2008 74 624 632 18584811 10.1055/s-2008-1034374

[7] He MF Liu L Ge W Shaw PC Liang R Wu LW But PPH Antiangiogenic activity of *Tripterygium wilfordii* and its terpenoids J. Ethnopharm 2009 121 61 68 10.1016/j.jep.2008.09.033 18996177

[8] Suyama TL Cao Z Murray TF Gerwick WH Ichthyotoxic brominated diphenyl ethers from a mixed assemblage of a red alga and cyanobacterium: structure clarification and biological properties Toxicon 2010 55 204 210 19638282 10.1016/j.toxicon.2009.07.020 PMC2813928

[9] Kita M Roy MC Siwu ER Noma I Takiguchi T Itoh M Yamada K Koyama T Iwashita T Uemura D Durinskiol A: a long carbon-chain polyol compound from the symbiotic dinoflagellate *Durinskia sp* Tetrahedron Lett 2007 48 3423 3427

[10] Bugni TS Harper MK McCulloch MW Reppart J Ireland CM Fractionated marine invertebrate extract libraries for drug discovery Molecules 2008 13 1372 1383 18596663 10.3390/molecules13061372 PMC2505051

[11] Bugni TS Richards B Bhoite L Cimbora D Harper MK Ireland CM Marine natural product libraries for high-throughput screening and rapid drug discovery J. Nat. Prod 2008 71 1095 1098 18505284 10.1021/np800184g PMC2533854

[12] Molinski TF Marine pyridoacridine alkaloids: structure, synthesis, and biological chemistry Chem. Rev 1993 93 1825 1838

[13] Molinski TF Fahy E Faulkner JD Van Duyne GD Clardy J Petrosamine, a novel pigment from the marine sponge *Petrosia sp* J. Org. Chem 1988 53 1341 1343

[14] Carroll AR Ngo AN Quinn RJ Redburn J Hooper JNA Petrosamine B, an inhibitor of the *Helicobacter pylori* enzyme aspartyl semialdehyde dehydrogenase from the Australian sponge *Oceanapia sp* J. Nat. Prod 2005 68 804 806 15921437 10.1021/np049595s

[15] Schmitz FJ Agarwal SK Gunasekera SP Schmidt PG Shoolery JN Amphimedine, new aromatic alkaloid from a pacific sponge, *Amphimedon sp.* Carbon connectivity determination from natural abundance ^13^C-^13^C coupling constants J. Am. Chem. Soc 1983 105 4835 4836

[16] Marshall KM Barrows LR Biological activities of pyridoacridines Nat. Prod. Rep 2004 21 731 751 15565252 10.1039/b401662a

[17] Marshall KM Matsumoto SS Holden JA Concepcion GP Tasdemir D Ireland CM Barrows LR The anti-neoplastic and novel topoisomerase II-mediated cytotoxicity of neoamphimedine, a marine pyridoacridine Biochem. Pharmacol 2003 66 447 458 12907244 10.1016/s0006-2952(03)00209-0

[18] Tasdemir D Marshall KM Mangalindan GC Concepcion GP Barrows LR Harper MK Ireland CM Deoxyamphimedine, a new pyridoacridine alkaloid from two tropical *Xestospongia* sponges J. Org. Chem 2001 66 3246 3248 11325300 10.1021/jo010153k

[19] de Guzman FS Carte B Troupe N Faulkner DJ Harper MK Concepcion GP Mangalindan GC Matsumoto SS Barrows LR Ireland CM Neoamphimedine: a new pyridoacridine topoisomerase II inhibitor which catenates DNA J. Org. Chem 1999 64 1400 1402

[20] Thale Z Johnson T Tenney K Wenzel PJ Lobkovsky E Clardy J Media J Pietraszkiewicz H Valeriote FA Crews P Structures and cytotoxic properties of sponge-derived bisannulated acridines J. Org. Chem 2002 67 9384 9391 12492342 10.1021/jo026459o

[21] Lindsay BS Barrows LR Copp BR Structural requirements for biological activity of the marine alkaloid ascididemin Bioorg. Med. Chem. Lett 1995 5 739 742

[22] Clement JA Kitagaki J Yang Y Sauceds CJ McMahon JB Discovery of new pyridoacridine alkloids from *Lissoclinum cf. badium* that inhibit the ubiquitin ligase activity of Hdm2 and stabilize p53 Bioorg. Med. Chem 2008 16 10022 10028 18977148 10.1016/j.bmc.2008.10.024 PMC2718708

[23] Charylulu GA McKee TC Ireland CM Diplamine, a cytotoxic polyaromatic alkaloid from the tunicate *Diplosoma sp* Tetrahedron Lett 1989 30 4201 4202

[24] McCarthy PJ Pitts TP Gunawardana GP Kelly-Borges M Pomponi SA Antifungal activity of meridine, a natural product from the marine sponge *Corticium sp* J. Nat. Prod 1992 55 1664 1668 1479383 10.1021/np50089a016

[25] Luedtke NW Hwang JS Glazer EC Gut D Kol M Tor Y Eilatin Ru(II) complexes display anti-HIV activity and enantiomeric diversity in the binding of RNA ChemBioChem 2002 3 766 771 12203975 10.1002/1439-7633(20020802)3:8<766::AID-CBIC766>3.0.CO;2-X

[26] Marshall KM Holden JA Koller A Kashman Y Copp BR Barrows LR AK37: the first pyridoacridine described capable of stabilizing the topoisomerase I cleavable complex Anti-Cancer Drugs 2004 15 907 913 15457132 10.1097/00001813-200410000-00012

[27] Westerfield M A Guide for the Laboratory use of Zebrafish (Danio rerio) 3rd ed University of Oregon Press Eugene, OR, USA 1995 385

[28] Nauduld LD Sandoval IT Chidester S Yost HJ Jones DA Adenomatous polyposis coli control of retinoic acid biosynthesis is critical for zebrafish intestinal development and differentiation J. Biol. Chem 2004 279 51581 51589 15358764 10.1074/jbc.M408830200

